# Detection of Plasmid-Mediated β-Lactamase Genes and Emergence of a Novel AmpC (CMH-1) in *Enterobacter cloacae* at a Medical Center in Southern Taiwan

**DOI:** 10.3390/jcm8010008

**Published:** 2018-12-20

**Authors:** Yee-Huang Ku, Mei-Feng Lee, Yin-Ching Chuang, Wen-Liang Yu

**Affiliations:** 1 Division of Infectious Disease, Department of Internal Medicine, Chi Mei Medical Center-Liouying, Tainan 710, Taiwan; althrisas@gmail.com; 2 Laboratory of Environmental Health, Research Center of Humanities and Technology, National University of Kaohsiung, Kaohsiung 811, Taiwan; mfphd@yahoo.com.tw; 3 Department of Medical Research, Chi Mei Medical Center, Tainan 710, Taiwan; chuangkenneth@hotmail.com; 4 Department of Intensive Care Medicine, Chi Mei Medical Center, Tainan 710, Taiwan; 5 Department of Medicine, School of Medicine, College of Medicine, Taipei Medical University, Taipei 110, Taiwan

**Keywords:** ACT, *ampC* gene, CMH-1, *Enterobacter cloacae*, plasmid

## Abstract

The plasmid-mediated extended-spectrum β-lactamases (ESBLs) and AmpC β-lactamases in *Enterobacter* spp. have increasingly been reported. In this study, we investigated the prevalence of the plasmid-mediated β-lactamases in *Enterobacter cloacae* from bloodstream isolates at a medical center in southern Taiwan. ESBL and *ampC* genes were detected by PCRs and DNA sequencing. Conjugation experiments were conducted to confirm the transferability of the genetic resistance trait. Among 41 non-repetitive blood isolates of cefuroxime-resistant *E. cloacae*, eight isolates exhibited ESBL phenotype confirmed by double-disk synergistic tests. Nearly all the strains were susceptible to carbapenems. The prevalence rate of the plasmid-mediated *bla*_ampC_ genes was 73% (30/41), including one *bla*_DHA-1_, one *bla*_MIR-6_, two novel *bla*_CMH-1_ genes and other *bla*_ACT-like_ genes. Coexistence of plasmid-mediated *bla*_ACT_ and ESBL genes (10 with *bla*_SHV-12_ and one with *bla*_CTX-M-3_) was observed. Successful transmissions of the *bla*_ACT_ and *bla*_CMH-1_ were demonstrated in some transconjugants. The inducible or derepressed CMH-1 had expanded activity of isolates versus ceftazidime. Enterobacterial repetitive intergenic consensus (ERIC)-PCR analysis and pulsotype showed distinct patterns suggesting non-clonal relationship. In conclusion, plasmid-mediated *bla*_ACT-like_ *ampC* genes in *E. cloacae* isolates have been highly prevalent in southern Taiwan and may continue genetic evolution, contributing to the complexities in antibiotic-resistant mechanisms.

## 1. Introduction

Resistance to β-lactam antibiotics is an emerging problem and β-lactamase production is the most common mechanism of antimicrobial resistance, especially in Gram-negative organisms [1]. In addition, the acquisition of resistance mechanisms including extended spectrum β-lactamases (ESBLs), plasmid-mediated AmpC β-lactamases and metallo-β-lactamases (MBLs) have been reported [2,3]. Among them, ESBLs that derive from genes for TEM-1, TEM-2, or SHV-1 by mutations have been reported worldwide in clinical *Enterobacteriaceae* and represent a significantly increasing problem of great concern, particularly for *Klebsiella pneumoniae* and *Escherichia coli* [4,5,6]. Chromosome-mediated AmpC enzymes confer resistance to ampicillin, cefazolin, and cefuroxime at low levels by repression of the promoter region of *ampC* gene [7]. In many bacteria, nonetheless, AmpC enzymes are inducible and can be expressed at high levels by derepressed mutation, and mediate resistance to broad-spectrum cephalosporins including cefotaxime, ceftazidime, and ceftriaxone. Furthermore, a chromosomal *ampC* gene could be transferred to a plasmid, and thus plasmid-mediated AmpC β-lactamases, although less common than ESBLs, have been found in several areas of the world [7]. In addition to humans, ESBLs and derepressed AmpC β-lactamases have been found in cefotaxime-resistant *E. coli* in the intestinal tract of healthy poultry [8]. Furthermore, ESBL-AmpC combinations were identified in clinical isolates of *Enterobacteriaceae* and could hamper the accurate detection of ESBL phenotypes by screening and confirmatory tests according to the recommendations of the Clinical and Laboratory Standards Institute [9].

Besides, multiple combinations of ESBLs and carbapenemases (like the KPC family and MBLs) have increased in *Enterobacter cloacae*, which might represent an emerging public health concern [10,11,12,13,14,15]. *E. cloacae* is a significant nosocomial pathogen that causes a wide range of infections, including pneumonia, and wound, urinary tract and bloodstream infections [16,17]. *E. cloacae* isolates are often multidrug-resistant and are capable of overproducing AmpC β-lactamases by induction and derepression of a chromosomal gene, or conferring the antibiotic resistance by the acquisition of a transfer-derepressed plasmid [7,14]. Of note, the coexistence of ESBL genes and *bla*_IMP-8_ is common in the carbapenem-non-susceptible *E. cloacae* isolates in China, including novel co-expressing *bla*_DNM-1_ and *bla*_IMP-26_ in the same strain [18]. These international events highlight the importance of continuous monitoring of the resistance trend and discovering the novel resistant mechanisms of *E. cloacae*.

In Taiwan, the emergence of MBLs in *Pseudomonas aeruginosa*, *E. cloacae* and *Citrobacter freundii* has been reported [19,20,21,22]. Meanwhile, the most commonly described carbapenem resistance in *Enterobacteriaceae* is the loss of major outer membrane porins together with the production of ESBLs and/or AmpC enzymes [23,24,25,26]. Meanwhile, combination of plasmid-mediated ESBLs (CTX-M-3 or SHV-types) and AmpC (CMY-2 or DHA-1) has been disseminated in *E. coli* and *K. pneumoniae* isolates in Taiwan [27,28]. ESBL-producing isolates of *E. cloacae* and *Serratia marcescens* have been identified in several hospitals [29].

Moreover, *E. cloacae* has emerged as an important hospital pathogen [26]. Multiple resistance mechanisms of MBLs, ESBLs and plasmid-mediated AmpC β-lactamases have been increasing in many bacteria among Taiwan hospitals thus resulting in diagnostic and therapeutic challenges; therefore, it is important to know the real prevalence rates of various resistance mechanisms in *E. cloacae* isolates producing MBLs, ESBL and/or AmpC β-lactamases. Based on our previous experience, clinical *E. cloacae* isolates with *ampC* genes might express resistance to cephalosporins limited to ampicillin, cefazolin and cefuroxime, but which would compromise therapeutic effects of broad-spectrum cephalosporins if *ampC* genes are inducible. However, for those *E. cloacae* isolates with resistance to broad-spectrum cephalosporins (for example, ceftazidime or ceftriaxone) when they are initially identified, the AmpC β-lactamases might be hyperproduced in a de-repressive mutant or be due to self-transference of a chromosomal *ampC* gene to a plasmid that mobilizes the *bla*_ampC_ without carrying its repressive promoter. Consequently, it is important to survey the prevalence of plasmid-mediated *ampC* gene in hospital bacterial populations, which might influence practicing physicians in selecting the empirical antimicrobial choices to mitigate therapeutic failure via a broad-spectrum cephalosporin. In addition, we have discovered two novel plasmid-mediated *ampC* genes (*bla*_MIR-6_ and *bla*_CMH-1_), coexisting with ESBLs, in *E. cloacae* isolates. Accordingly, the aims of our study were to investigate the prevalence of plasmid-mediated resistance genes that might produce MBLs, ESBLs and/or AmpC β-lactamases, including CMH-1 and MIR-6, in a collection of *E. cloacae* bloodstream infection isolates from a single institute.

## 2. Material and Methods

### 2.1. Clinical Isolates and Antimicrobial Susceptibility Testing

Non-repetitive clinical isolates of cefuroxime-resistant *E. cloacae* isolates from bloodstream infection were collected from Chi Mei Medical Center in southern Taiwan during 2007–2011. The isolates were frozen at −70 °C in Luria Bertani broth with 15% glycerol prior to testing. ESBL phenotype was confirmed by double-disk synergy test (DDST) [30]. Minimal inhibitory concentrations (MICs) were determined by using commercially available dry plates (Sensititre NHRIGN9; TREK Diagnostic Systems, Cleveland, OH) and were interpreted according to the guidelines of the Clinical Laboratory Standards Institute [31]. *E. coli* ATCC 25922 was used as a drug-susceptible strain of quality control. *K. pneumonia* ATCC 700603 was used as a quality control strain for ESBL detection.

### 2.2. DNA Manipulation, PCR Amplification and Sequencing

Plasmids from the isolates were extracted by alkaline lysis procedure [32]. The plasmid DNA was used as a template under standard PCR conditions with a series of primers designed for the detection of the class A β-lactamase genes including *bla*_KPC_, *bla*_GES_, *bla*_SME_, *bla*_IMI_; class B β-lactamase genes including *bla*_IMP_, *bla*_VIM_, *bla*_NDM-1_; *ampC* genes; and class D β-lactamase genes [33,34,35]. All oligonucleotide primers used in this study were tabulated in Table 1 [36,37,38,39]. Moreover, after initial screening with *EBC* (a family of *ampC* genes) primers (shown in Table 1) and subsequent PCR product DNA sequencing, specific novel primers were designed for PCR amplification, cloning and entire DNA sequencing analysis to confirm *bla*_CMH-1_ and *bla*_MIR-6_ genes by using *CMH-1* F (5′ ATGATGACAAAATCCCTAAGCTG 3′) and *CMH-1* R (5′ TTACTGTAGCGCGTCGAGGATA 3′) as well as *MIR-6* F (5′ ATGATGACAAAATCCCTAAGCTG 3′) and *MIR-6* R (5′ TTACTGCAGCGCGTCGACG 3′) respectively. The Basic Local Alignment Search Tool (BLAST) website at the National Center for Biotechnology Information (NCBI) was applied for searching, analyzing and aligning sequences (www.ncbi.nlm.nih.gov). The PCR products were sequenced using a forward primer and a reverse primer for paired matching. The novel *CMH-1* and *MIR-6* primers were designed based on the reference sequences from chromosomal *ampC* gene in *E. cloacae* ATCC 13047 strain (accession number YP_003611068) and plasmid-mediated *MIR-5* *ampC* gene from *K. pneumoniae* 801 EBC801 strain (accession number NG_049306) respectively. The sizes of PCR products of *CMH-1* and *MIR-6* were both 1146 bp. In addition, the PCR-NheI method was used to discriminate SHV-type ESBLs from non-ESBLs. PCR-NheI restriction analysis suggested by Nuesch-Inderbinen et al. employs a PCR-restriction fragment length polymorphism method, using a restriction enzyme of NheI to detect a point mutation of Gly238Ser within the sequences of PCR products, which might distinguish the majority of the SHV-derived ESBL variants from the *SHV-1* gene [40]. 

### 2.3. Plasmid Conjugation Experiments and Southern Hybridization

Conjugation experiments were performed by the filter mating method and a rifampin-resistant strain *E. coli* J53 was used as the recipient strain [41]. Plasmid analysis was performed by electrophoresis at 100 V for 50 min in 1% agarose gel. Southern hybridization was carried out with a digoxigenin (DIG)-labeled probe targeting for the *bla*_MIR/ACT_ gene (closely related to chromosomal *EBC* family *ampC* gene, Table 1) using a DIG system [37].

### 2.4. Pulsed-Field Gel Electrophoresis (PFGE)

Genomic DNA of *E. cloacae* isolates was extracted as described previously [42]. Enterobacterial repetitive intergenic consensus (ERIC)-PCR analysis was used to determine the genomic relatedness between isolates [43]. PFGE was performed with a CHEF Mapper XA System (Bio-Rad Laboratories) using *Xba*I (Bio-Rad) for DNA digestion [42]. Cluster analyses of pulsotypes were performed by the UPGMA (Unweighted Pair Group Method with Arithmetic mean) algorithms and were compared using the BioNumerics program, which is commercial software purchased from the bioMérieux company (Applied Maths Inc., Austin, TX, USA). Similarity coefficients were calculated by using the Dice algorithm, a set of statistical tools in the BioNumerics program [44,45].

## 3. Results

### 3.1. Bacterial Strains and Antimicrobial Susceptibility Profiles

Forty-one cefuroxime-resistant *E. cloacae* isolates from bloodstream infections were collected and tested with MICs shown in Table 2. MICs for amikacin ranged from ≤4 mg/L to 32 mg/L (MIC_50_ = 4 mg/L and MIC_90_ = 8 mg/L). MICs for ciprofloxacin ranged from ≤0.06 to >2 mg/L (MIC_50_ = 0.06 mg/L and MIC_90_ > 2 mg/L). MICs for tigecycline ranged from ≤0.25 to >2 mg/L (MIC_50_ = 1 mg/L and MIC_90_ > 2 mg/L). MICs for colistin ranged from 0.5 to >2 mg/L (MIC_50_ = 1 mg/L and MIC_90_ > 2 mg/L). MICs for ertapenem ranged from 0.25 mg/L to 2 mg/L (MIC_50_ = 0.5 mg/L and MIC_90_ = 1 mg/L). MICs for imipenem ranged from 0.25 to 2 mg/L (MIC_50_ = 0.5 mg/L and MIC_90_ = 1 mg/L). MICs for doripenem ranged from ≤0.25 mg/L to 2 mg/L (MIC_50_ = 0.25 mg/L and MIC_90_ = 0.25 mg/L). MICs for meropenem ranged from 0.25 mg/L to 0.5 mg/L (MIC_50_ = 0.25 mg/L and MIC_90_ = 0.25 mg/L). Eight isolates were confirmed to exhibit the ESBL phenotype by using DDST.

### 3.2. Detection of β-Lactamase Genes on Plasmid and Antibiograms

The PCR methods using specific primers as presented in Table 1 did not identify any isolate containing a class A carbapenemase gene (such as *bla*_KP_C and *bla*_GES_) or a class B MBLs gene (such as *bla*_IMP_, *bla*_VIM_, and *bla*_NDM_). 

The plasmid-mediated *ampC* genes were found in 30 *E. cloacae* isolates (73%, 30/41), according to PCR analysis based on plasmid DNA preparation as templates. Among the 30 isolates with plasmid-mediated *bla*_ampC_, coexisting *bla*_SHV-12_ (*n* = 10), *bla*_TEM-1_(*n* = 7) and *bla*_CTX-M-3_ (*n* = 1) were identified (Table 3). Among the seven isolates with *bla*_TEM-1_, four isolates also harbored a *bla*_ampC_ and a *bla*_SHV-12_. Among ten isolates with a *bla*_SHV-12_ gene, seven isolates exhibited ESBL phenotype. The eight isolates with ESBL phenotype were contributed by SHV-12 (*n* = 7) and CTX-M-3 (*n* = 1). Moreover, two novel plasmid-mediated *bla*_CMH-1_ (accession number JQ673557, *n* = 2) and *bla*_MIR-6_ (accession number JQ664733, *n* = 1) were identified and other *bla*_ampC_ genes were unnamed *ACT*-like *ampC* genes with various identities close to chromosomal *ACT-9* or *ACT-2* genes belonging to *EBC* family. The *bla*_CMH-1_ gene has high identification of 99% to a chromosomally intrinsic *ampC* gene in *E. cloacae* ATCC 13047 strain (accession number YP_003611068), 88% identification to a chromosomal *ACT-9* gene in *Pantoea agglomerans* (accession number YP_004712370), and 87% identification to a chromosomal *ACT-2* gene in *Enterobacter asburiae* (accession number CAJ28994) (Supplementary Figures S1–S4). The *bla*_MIR-6_ gene has high identification of 99% to a plasmid-mediated *bla*_MIR-5_ *ampC* gene found in *K. pneumoniae* 801 EBC801 strain (accession number NG_049306). In addition to *MIR*/*ACT* gene, a plasmid-mediated *ampC* gene of *DHA-1* was identified in this study (*n* = 1).

The plasmid-mediated *ampC* genotypes or those coexisting with ESBL genes were difficult to predict by classification of antimicrobial resistance phenotypes (antibiograms). The antibiotic resistance codes were designed by two components: part I profile was based on β-lactamase phenotype (resistance to ceftazidime, cefotaxime, ceftriaxone and cefepime); and part II profile was based on co-resistance to imipenem, ciprofloxacin and aminoglycosides (Table 3). The most common antibiogram of isolates with a plasmid-mediated *ampC* gene was type I resistance code, indicating hyperproduction of AmpC β-lactamases. However, the second common antibiogram of isolates with a plasmid-mediated *ampC* gene was type III resistance code, indicating low-level or repressive AmpC production, which was difficult to differentiate from those isolates without a plasmid-mediated *ampC* gene. The resistance codes V to VII might indicate isolates coexisting with ESBL genes, particularly with emphasis on resistance to cefepime (Table 3). 

### 3.3. Plasmid Profiles and Location of Resistance Gene

**Figure 1:**
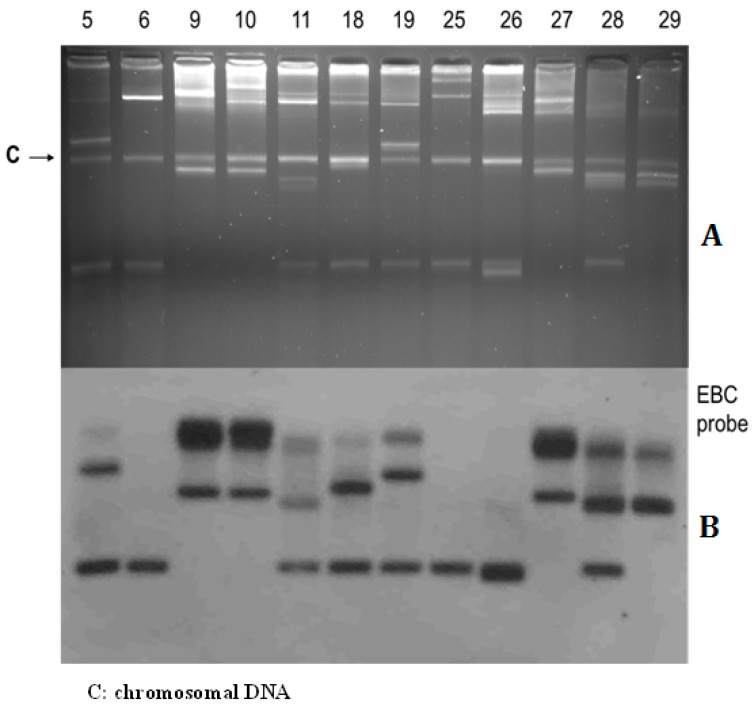
(**A**) Plasmid analysis shows different profiles of *E. cloacae* isolates and (**B**) southern hybridization analysis shows *bla*_ACT-like_ genes hybridizing with the *Enterobacter cloacae* using *EBC* primer-specific probe. Note. 5, EntC-5 strain; 6, EntC-6 strain; 28, EntC-28 strain; 29, EntC-29 strain.

Plasmid profiles were studied in the 41 isolates of *E. cloacae*. Plasmid analysis revealed different plasmid profiles (partially shown in Figure 1A). Meanwhile, *bla*_ACT-like_ *ampC* genes encoded on plasmids of 30 *E. cloacae* isolates were also identified by hybridizing with the *EBC* primer-specific probe (partially shown in Figure 1B). 

**Figure 2:**
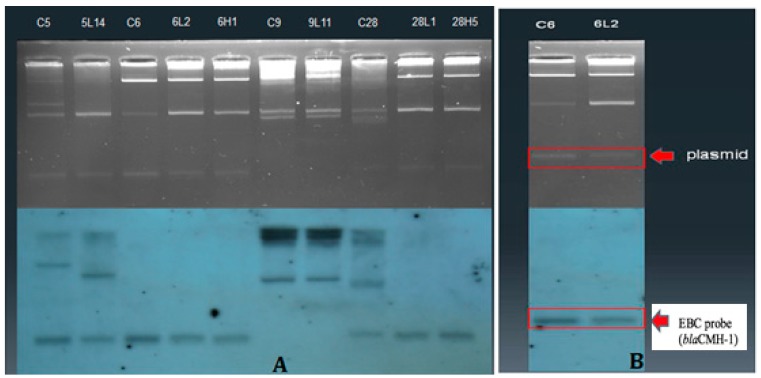
(**A**) Plasmid analysis (upper) and Southern hybridization with *EBC* primer-specific probe (lower) are showing on plasmids encoding *bla*_ACT-like_ genes of *Enterobacter cloacae* parental strains and *Escherichia coli* transconjugants. (**B**) Plasmid analysis (upper) and Southern hybridization with the *EBC* probe (lower) are showing on plasmids encoding *bla*_CMH-1_ of donor C6 (EntC-6) strain and transconjugant 6L2. Transconjugant 6L2 was the second colony on the filter mating plate from parental EntC-6 strain at low concentration of rifampin in the conjugation experiment. The *bla*_ACT-like_ genes were revealed by PCR and DNA sequencing. The *bla*_CMH-1_ was confirmed by PCR, cloning and DNA sequencing.

Some of the AmpC-producing plasmids, for example, in strains EntC-5, EntC-28, and EntC-6 that harboring *bla*_CMH-1_, could be transferred into the transconjugant strains of *E. coli* J53 in the conjugated experiments (Figure 2). PCR on plasmid templates from parental and transconjugant strains using specific *EBC*, *CMH-1* and *MIR-6* primers and subsequent DNA sequencing and cloning analysis revealed that *bla*_ACT-like_ genes (in EntC-5 and EntC-28 strains) and *bla*_CMH-1_ (in EntC-6 strain) were encoded on plasmids of parental and transconjugant strains. Nonetheless, *bla*_MIR-6_ was only found in EntC-29 parental strain but not in its transconjugant strain (data not shown).

The MICs of *E. cloacae* EntC-6 parental strain and its transconjugant showed high-level activities of the plasmid-mediated CMH-1 against ceftazidime, cefotaxine and ceftriaxone (Table 4). The colistin resistance gene was not successfully conjugated into *E. coli* J53 recipient. The *E. cloacae* parental strains (EntC-5 and EntC-28) showed relatively low-level MICs for ceftazidime, cefotaxine and ceftriaxone (0.5–4 mg/L), whereas their transconjugants (5L14, 5H15, 28L1 and 28H5) exhibited higher levels of MIC for ceftazidime (≥256 mg/L) and variable levels of MIC for cefotaxime and ceftriaxone (16–256 mg/L). The transference of plasmid-mediated *bla*_MIR-6_ of *E. cloacae* EntC-29 has failed in the conjugation experiments.

### 3.4. Molecular Typing for Genomic DNA

**Figure 3:**
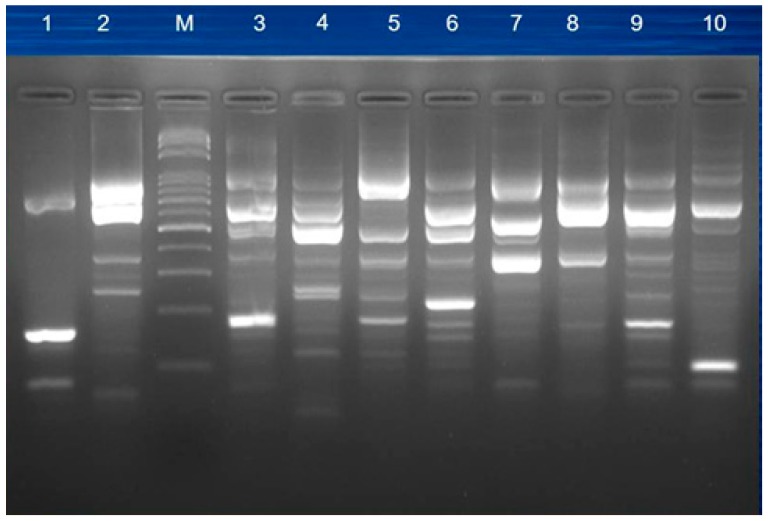
The electrophoresis result by the ERIC-PCR reaction system. M: 100 bp DNA ladder marker (Protech, Taipei, Taiwan).

**Figure 4:**
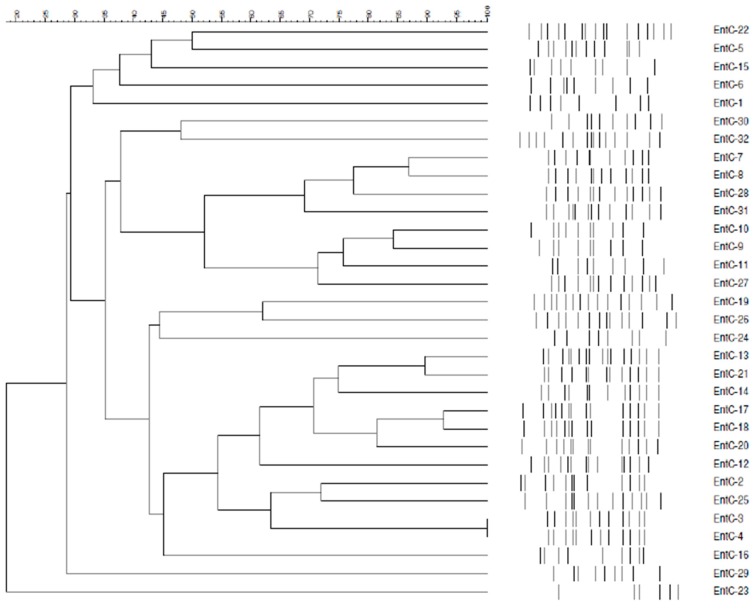
Genomic cluster analysis and pulsotype profiles of PFGE for 32 bloodstream infection *E. cloacae* isolates, including 30 isolates with plasmid-mediated *ampC* genes and 2 isolates without plasmid-mediated *ampC* genes (EntC-23 and EntC-24).

The results of ERIC-PCR patterns were very heterogeneous (partially shown in Figure 3), suggesting non-clonal relationship of the studied isolates. In addition, based on PFGE pulsotype patterns, 32 selected isolates were separated into different groups, also suggesting an unrelated genetic relationship (Figure 4).

## 4. Discussion

Infections caused by *E. cloacae* are difficult to treat as the majority of isolates exhibit varying degrees of β-lactamase-mediated resistance to most of the third-generation cephalosporins. The degree of resistance of an isolate with low levels of AmpC production is inducible to high-level resistance by an initially susceptible cephalosporin, which itself might play a role of strong inducer of AmpC production. In fact, they are capable of overproducing AmpC β-lactamases by induction of a β-lactam antibiotic, by derepression of a chromosomal *ampC* gene, or by the acquisition of a transferable *ampC* gene on the plasmids, thus conferring resistance to the broad-spectrum antibiotics except for fourth-generation cephalosporins [46,47]. Some *E. cloacae* strains are now both ESBL and AmpC co-producers and could therefore confer resistance to both third- and fourth-generation cephalosporins [16].

Among a collection of 117 Malaysian isolates of *Enterobacter* species, 39% of isolates were resistant to cefotaxime and ceftriaxone, 24% were resistant to ceftazidime, 8.5% were resistant to cefepime, and one isolate was resistant to meropenem. Chromosomal *EBC* family gene was amplified from 36 (47%) *E. cloacae* and three (25%) *E. asburiae* [48]. A study of *E. cloacae* isolates from central Taiwan reported a susceptibility rate of 53% to ceftazidime [9]. Focusing on cefuroxime-resistant *E. cloacae* isolates from southern Taiwan, we found a higher prevalence rate of plasmid-mediated *ampC* (73%) and ESBL genes (27%). Amikacin and carbapenems remained the most active compounds against these isolates (resistance rates, <10%), followed by colistin and cefepime (resistance rates, <20%), ciprofloxacin and tigecycline (resistance rates, <25%), and piperacillin-tazobactam (resistance rate, <30%). Aztreonam, ceftazidime and cefotaxime showed higher resistance rates of 54–56%. The ACT-like β-lactamases generally showed the highest activities against cefuroxime, cefoxitin, cefazolin and ampicillin. Nonetheless, the transconjugants with de-repressed ACT-like AmpC exhibited high-level MICs for ceftazidime, suggesting that substantial instances of the plasmid-mediated *bla*_ACT-like_ genes were repressed or not fully expressed in the parental strains. The SHV-12 and CTX-M-3 also converted resistance of *E. cloacae* to aztreonam, ceftazidime and cefotaxime. Together with ESBL and some decrease of fluoroquinolone activities, AmpC-producing *Enterobacter* bloodstream infections will pose substantial therapeutic challenges to physicians. 

The chromosomal *ampC* genes of *EBC* family (*MIR*-and *ACT*-types) have been identified in *E. cloacae* from central Taiwan [9]. MIR-1 and ACT-1, first identified in *K. pneumoniae* isolates, are the plasmid-mediated AmpC-type β-lactamase that originated from chromosome of *E. cloacae* [49,50]. In a recent study of 53 *E. cloacae* bloodstream isolates from Shanghai, China, 18 (34%) were plasmid-mediated AmpC producers with a predominance of MIR/ACT types [51]. In the current study of 41 *E. cloacae* bloodstream isolates from southern Taiwan, 30 (73%) were plasmid-mediated ACT-like producers, including two isolates (EntC-6 and EntC-32) with a novel plasmid-mediated *CMH-1* of *EBC* variant that showed a high level of resistance to ceftazidime (MIC, 128–256 mg/L) in both parental and transconjugants, suggesting inducible/derepressed and transferable characteristics of the *bla_CMH-1_* gene. The *E. cloacae* EntC-29 strain harboring plasmid-mediated *bla*_MIR-6_ probably showed a repressed phenotype of low-level MICs to ceftazidime, cefotaxime and ceftriaxone.

As for the failure of transference for *bla*_MIR-6_ in conjugation experiments, the derepressed or inducible phenotype of MIR-6 could not be demonstrated in the study. However, the presence of *bla*_MIR-like_ *ampC* genes on the plasmids among *K. pneumoniae* and *E. cloacae* highlights the capability of mobility of *bla*_MIR_ between different germs of *Enterobacteriaceae*. Furthermore, a novel plasmid-mediated *CMH-2* *ampC* gene with a sequence similarity of 98.6% to *CMH-1* was recovered from two clinical *K. pneumoniae* isolates in India, suggesting continuous evolution and spreading of the *bla*_CMH_ resistance trait [52].

Unlike CTX-M-type enzymes frequently predominating in *E. coli*, *K. pneumoniae*, *Proteus mirabilis* and *S. marcescens* [39,53,54,55], SHV-12 is the major type of ESBL found in *E. cloacae* [56]. IS26 was recognized to play a role in the dissemination of *bla*_SHV-12_ by the transposons between different plasmids in multidrug-resistant *E. cloacae* isolates [57]. A previous report from central Taiwan identified 15.5% of *E. cloacae* isolates as ESBL-producers with a predominance of SHV-12 [56]. In the current study, although 11 (27%) isolates harbored ESBL genes (10 *SHV-12* and one *CTX-M-3*), only eight isolates exhibit ESBL phenotype by DDST, which expression could probably be hampered by the coexistence of plasmid-mediated ACT-like enzymes in three isolates with *bla*_SHV-12_ and additional DHA-1 in one isolate.

The plasmid-mediated *bla*_ACT_ genes, including *bla*_CMH-1_ gene, in *E. cloacae* might substantially enhance the capability of transmission of the resistance trait by different plasmid dissemination among clinical isolates with genetic diversity as shown by plasmid analysis and genomic typing methods. The ERIC-PCR amplification of *E. cloacae* isolates in the current study revealed different electrophoresis banding patterns and PFGE showed multiple pulsotype profiles, which provided more discriminative DNA patterns of the study population. The above studies conclude that the resistance genes in *E. cloacae* were involved in horizontal spreads of plasmids but not chromosomally clonal dissemination in of our study setting.

The limitations of the work include a rather small sample size of only 41 strains and a focus on bloodstream infection at a single institute, so that our conclusion might not be generally applicable to infections at different specific sites or to other hospital settings. However, routine surveillance and monitoring of the genetic evolution for antimicrobial resistance are important.

## 5. Conclusions

In the present study, high occurrence rates of plasmid-mediated *ampC* genes have been identified. Evidence of multiple plasmid-mediated *ampC* and ESBL genes in a single strain was identified. We demonstrated that the *ACT-like* and *CMH-1* *ampC* genes are able to mobilize to different plasmids, some of which could further be self-transferred to *E. coli* recipient strains. The plasmid-mediated *ACT-like* *ampC* genes and coexistence with ESBL genes (mainly *bla*_SHV-12_) in *E. cloacae* isolates have been highly prevalent in southern Taiwan, and together with emergence of novel *CMH-1* and *MIR-6*, might contribute to continuous evolution and complexity of antibiotic resistance mechanisms of the isolates.

The diversity of resistant patterns and mechanisms of *E. cloacae* isolates, capable of carrying multiresistant genes of the plasmid, self-transference of plasmid and high prevalence of plasmid-mediated *ampC* genes, including novel evolution of *CMH-1* and *MIR-6* genes, as observed in this study, might contribute to the broad dissemination of resistance traits among *E. cloacae* isolates in hospital environments. The impact of the novel findings will highlight the need to change physicians’ habits of empirical antibiotic prescription when an *E. cloacae* strain is identified in the bloodstream infection before the results of the antimicrobial susceptibilities are available. Empirical extended-spectrum cephalosporins should not be recommended in such hospital environments. New β-lactam/β-lactamase inhibitor combinations, ciprofloxacin or carbapenems, as alternative options, might be the drug of choice, as the selective pressure of such compounds on antimicrobial resistance would still be low in the hospital.

## Figures and Tables

**Table 1:** Oligonucleotide primers used in this study.

Genes	Primers	Oligonucleotide Sequence (5′–3′)	Reference
Class A Carbapenemases			
SME	SME-F	AGATAGTAAATTTTATAG	[36]
	SME-R	CTCTAACGCTAATAG	
IMI	IMI-F	ATAGCCATCCTTGTTTAGCTC	[36]
	IMI-R	TCTGCGATTACTTATCCTC	
KPC	KPC-F	ATGTCACTGTATCGCCGTCT	[36]
	KPC-R	TTTTCAGAGCCTTACTGCCC	
GES	GES-F	GTTTTGCAATGTGCTCAACG	[36]
	GES-R	TGCCATAGCAATAGGCGTAG	
Class B metallo-β-lactamases			
IMP-1	IMP-1-F	TGAGCAAGTTATCTGTATTC	[36]
	IMP-1-R	TTAGTTGCTTGGTTTTGATG	
IMP-2	IMP-2-F	GGCAGTCGCCCTAAAACAAA	[36]
	IMP-2-R	TAGTTACTTGGCTGTGATGG	
VIM-1	VIM-1-F	TTATGGAGCAGCAACCGATGT	[36]
	VIM-1-R	CAAAAGTCCCGCTCCAACGA	
VIM-2	VIM-2-F	AAAGTTATGCCGCACTCACC	[36]
	VIM-2-R	TGCAACTTCATGTTATGCCG	
NDM	NDM-F	TCTCGACAATGCCGGGTTT	In this study
	NDM-R	GAGATTGCCGAGCGACTT	
AmpC β-lactamases			
CMY-2	AmpC-1B	TTTTCAAGAATGCGCCAGGC	In this study
	AmpC-1C	CTGCTGCTGACAGCCTCTTT	
DHA-1	DHA-1A	CTGATGAAAAAATCGTTATC	In this study
	DHA-1B	ATTCCAGTGCACTCAAAATA	
MOX	MOXMF	GCTGCTCAAGGAGCACAGGAT	[37]
	MOXMR	CACATTGACATAGGTGTGGTGC	
CIT	CITMF	TGGCCAGAACTGACAGGCAAA	[37]
	CITMR	TTTCTCCTGAACGTGGCTGGC	
DHA	DHAMF	AACTTTCACAGGTGTGCTGGGT	[37]
	DHAMR	CCGTACGCATACTGGCTTTGC	
ACC	ACCMF	AACAGCCTCAGCAGCCGGTTA	[37]
	ACCMR	TTCGCCGCAATCATCCCTAGC	
FOX	FOXMF	AACATGGGGTATCAGGGAGATG	[37]
	FOXMR	CAAAGCGCGTAACCGGATTGG	
EBC	EBCMF	TCGGTAAAGCCGATGTTGCGG	[37]
	EBCMR	CTTCCACTGCGGCTGCCAGTT	
CMH-1	CMH-1F	ATGATGACAAAATCCCTAAGCTG	In this study ^a^
	CMH-1R	TTACTGTAGCGCGTCGAGGATA	
MIR-6	MIR-6F	ATGATGACAAAATCCCTAAGCTG	In this study ^a^
	MIR-6R	TTACTGCAGCGCGTCGACG	
Class D Oxacillinases			
OXA-48	OXA-48-F	GATGTGTCATAGTATTCGTCG	[38]
	OXA-48-R	TCACAACAACTAAAAGCACTG	
OXA-1	OXA-1A	TCAACTTTCAAGATCGCA	[38]
	OXA-1B	GTGTGTTTAGAATGGTGA	
OXA-9	OXA-9A	TTCGTTTCCGCCACTCTCCC	[38]
	OXA-9B	ACGAGAATATCCTCTCGTGC	
ESBL genes			
SHV	SHV specific F	GATCCACTATCGCCAGCAGG	In this study
	SHV specific R	ACCACAATGCGCTCTGCTTTG	
CTX-M-1 group	CTX-M-1F	GGTTAAAAAATCACTGCGTC	[39]
	CTX-M-1R	TTGGTGAGATTTTAGCCGC	
CTX-M-2 group	CTX-M-2F	TGGGTTACGATTTTCGCCGC	[39]
	CTX-M-2R	TGGGTTACGATTTTCGCCGC	
CTX-M-9 group	CTX-M-9F	ATGGTGACAAAGAGAGTGCA	[39]
	CTX-M-9R	CCCTTCGGCGATGATTCTC	
TEM	TEM-F	ATGAGTATTCAACATTTCCG	[39]
	TEM-R	CCAATGCTTAATCAGTGAGG	

Note. ^a^ for cloning and entire *ampC* DNA sequencing.

**Table 2:** Antibiotic susceptibilities of the bloodstream *E. cloacae* isolates.

	MIC (mg/L)				
	*R* Criteria	Range	MIC_50_	MIC_90_	*R* (%)
Amikacin	≥64	≤4–32	≤4	8	0
Amoxicillin/clavulanic acid	≥32	≤8–32	>32	>32	93
Ampicillin	≥32	≤2–16	>16	>16	95
Aztreonam	≥16	≤1–16	4	>16	54
Cefazolin	≥8	≤2–32	>32	>32	95
Cefepime	≥16	≤1–16	≤1	16	17
Cefotaxime	≥4	≤1–32	16	>32	54
Cefoxitin	≥32	≤4–16	>16	>16	95
Ceftazidime	≥16	≤1–16	4	>16	54
Cefuroxime	≥32	4–16	>16	>16	95
Ciprofloxacin	≥4	≤0.06–2	≤0.06	>2	20
Colistin	>2 ^a^	≤0.5–2	1	>2	12
Doripenem	≥4	≤0.25–2	0.25	0.25	0
Ertapenem	≥2	≤0.25–2	0.5	1	7
Gentamicin	≥16	≤1–8	1	>8	24
Imipenem	≥4	≤0.25–2	0.5	1	0
Meropenem	≥4	≤0.25–0.5	0.25	0.25	0
Piperacillin/tazobactam	≥128	≤4–64	8	>64	29
Tigecycline	>2 ^b^	≤0.25–2	1	>2	24

Note. MIC: minimal inhibitory concentration; R: resistance; ^a^ According to the European Committee on Antimicrobial Susceptibility Testing (EUCAST) breakpoint; ^b^ EUCAST breakpoint.

**Table 3:** Plasmid-mediated β-lactamase genes were found in 30 of 41 *E. cloacae* bloodstream isolates.

Strain No.	Plasmid-Mediated β-Lactamase Gene(s)	Resistance Profiles (I) CAZ/CTX/CRO/FEP	Resistance Profiles (II) IPM/CIP/GM/AN	Resistance Code
EntC-1	ACT-like	R/R/R/S (A)	S/S/S/S (a)	Aa (I)
EntC-2	ACT-like	R/R/R/S (A)	S/S/R/S (b)	Ab (II)
EntC-3	ACT-like/TEM-1/SHV-12 ^a^	R/R/R/S (A)	S/S/S/S (a)	Aa (I)
EntC-4	ACT-like	S/S/S/S (B)	S/S/S/S (a)	Ba (III)
EntC-5	ACT-like	S/S/S/S (B)	S/S/S/S (a)	Ba (III)
EntC-6	TEM-1/CMH-1	R/R/R/S (A)	S/S/R/S (b)	Ab (II)
EntC-7	ACT-like	R/R/R/S (A)	S/S/S/S (a)	Aa (I)
EntC-8	ACT-like	R/R/R/S (A)	S/S/S/S (a)	Aa (I)
EntC-9	ACT-like/TEM-1/SHV-12	R/R/R/S (A)	S/R/R/S (c)	Ac (IV)
EntC-10	TEM/ACT-like	R/R/R/R (C)	S/R/R/S (c)	Cc (V)
EntC-11	ACT-like/TEM-1/SHV-12 ^a^	R/R/R/R (C)	S/R/S/S (d)	Cd (VI)
EntC-12	ACT-like/TEM-1/SHV-12 ^a^	R/R/R/S (A)	S/S/R/S (b)	Ab (II)
EntC-13	ACT-like/TEM-1/SHV-12 ^a^	R/R/R/S (A)	S/S/S/S (a)	Aa (I)
EntC-14	ACT-like	R/R/R/S (A)	S/S/S/S (a)	Aa (I)
EntC-15	ACT-like	S/S/S/S (B)	S/S/S/S (a)	Ba (III)
EntC-16	ACT-like/TEM-1	R/R/R/S (A)	S/S/R/S (b)	Ab (II)
EntC-17	ACT-like/SHV-12 ^a^	R/R/R/R (C)	S/R/R/S (c)	Cc (V)
EntC-18	ACT-like/SHV-12 ^a^	R/R/R/R (C)	S/R/S/S (d)	Cd (VI)
EntC-19	ACT-like	R/R/R/S (A)	S/S/S/S (a)	Aa (I)
EntC-20	ACT-like/SHV-12 ^a^	R/R/R/R (C)	S/R/R/S (c)	Cc (V)
EntC-21	ACT-like	S/S/S/S (B)	S/S/S/S (a)	Ba (III)
EntC-22	ACT-like	R/R/R/S (A)	S/S/S/S (a)	Aa (I)
EntC-23	Not identified	S/S/S/S (B)	S/S/S/S (a)	Ba (III)
EntC-24	Not identified	S/S/S/S (B)	S/S/S/S (a)	Ba (III)
EntC-25	ACT-like/DHA-1/SHV-12	R/R/R/R (C)	S/S/R/S (b)	Cb (VII)
EntC-26	ACT-like/CTX-M-3 ^a^	R/R/R/R (C)	S/R/R/S (c)	Cc (V)
EntC-27	ACT-like	R/R/R/S (A)	S/R/S/S (d)	Ad (VIII)
EntC-28	ACT-like	S/S/S/S (B)	S/S/S/S (a)	Ba (III)
EntC-29	MIR-6	S/S/S/S (B)	S/S/S/S (a)	Ba (III)
EntC-30	ACT-like/SHV-12	S/S/S/S (B)	S/S/S/S (a)	Ba (III)
EntC-31	ACT-like	S/S/S/S (B)	S/S/S/S (a)	Ba (III)
EntC-32	CMH-1	R/R/R/S (A)	S/S/S/S (a)	Aa (I)
EntC-33 to EntC-41	Not identified	S/S/S/S (B)	S/S/S/S (a)	Ba (III)

Note. EntC-23, EntC-24 and Ent-33 to EntC-41strains did not harbor plasmid-mediated *ampC* genes. CAZ, ceftazidime; CTX, cefotaxime; CRP, ceftriaxone; FEP, cefepime; IMP, imipenem; CIP, ciprofloxacin; GM, gentamicin; AN, amikacin; R, resistance. The number and percentage of resistance code revealed type I (9, 22%), II (4, 10%), III (19, 46%), IV (1, 2%), V (4, 10%), VI (2, 5%), VII (1, 2%) and VIII (1, 2%). ^a^: positive for ESBL phenotype by double-disk synergy test.

**Table 4:** Minimal inhibitory concentrations of *Enterobacter cloacae* (including parental, recipient and transconjugant strains) with a novel plasmid-mediated *ampC* gene.

Strain	Source	AmpC	CAZ	CTX	CRO	FEP	IPM	CIP	GM	TGC	CL
*E. coli* J53			2	0.25	0.25	0.06	2	2	1	1	0.5
EntC-6	Blood	CMH-1	128	64	128	2	0.5	1	>64	1	>16
6L2		CMH-1	256	32	16	8	2	<0.03	>64	1	1
EntC-5	Blood	ACT-like	4	2	4	1	0.5	<0.03	1	2	1
5L14		ACT-like	>256	128	256	8	0.5	0.5	>64	8	1
5H15		ACT-like	256	32	16	8	4	4	>64	1	1
EntC-28	Blood	ACT-like	1	0.5	1	0.13	1	<0.3	1	1	2
28L1		ACT-like	256	16	16	8	2	2	>64	1	1
28H5		ACT-like	256	16	16	8	4	4	>64	2	1
EntC-32	Blood	CMH-1	128	128	128	0.5	1	0.25	1	1	>16
EntC-29	Blood	MIR-6	0.13	0.13	1	0.03	1	<0.31	1	1	1
ATCC *E. coli* 25922			1	0.13	0.13	0.63	0.25	<0.03	2	0.25	1

Note. *E. coli* J53: recipient; EntC-6, EntC-5, EntC-28: parental strains of *E. cloacae*; 6L2, 5L14, 5H15, 28L1, 28H5: transconjugants of *E. coli*; 6L2 was the second colony on the filter mating plate from parental EntC-6 strain at low concentration of rifampin in the conjugation experiment; 28H5 was the fifth colony on the filter mating plate from EntC-28 strain at high concentration of rifampicin in the conjugation experiment; CAZ: ceftazidime; CTX: cefotaxime; CRO: ceftriaxone; FEP: cefepime; IPM: imipenem; CIP: ciprofloxacin; GM: gentamicin; TGC: tigecycline; CL: colistin.

## Supplementary Materials

The following are available online at http://www.mdpi.com/2077-0383/8/1/8/s1, Figure S1: Complete DNA sequence of *bla*_CMH-1_ gene (accession number JQ673557), Figure S2: The *bla*_CMH-1_ gene has high identities of 99% to a chromosomally intrinsic *ampC* gene in *Enterobacter cloacae* ATCC 13047 strain (accession number YP_003611068), Figure S3: The *bla*_CMH-1_ gene has 88% identities to a chromosomal *ACT-9* gene in *Pantoea agglomerans* (accession number YP_004712370), Figure S4: The *bla*_CMH-1_ gene has 87% identities to a chromosomal *ACT-2* gene in *Enterobacter asburiae* (accession number CAJ28994).

## References

[1] Dallenne C., da Costa A., Decre D., Favier C., Arlet G. (2010). Development of a set of multiplex PCR assays for the detection of genes encoding important β-lactamases in *Enterobacteriaceae*. J. Antimicrob. Chemother..

[2] Manchanda V., Singh N.P. (2003). Occurrence and detection of AmpC β-lactamases among Gram-negative clinical isolates using a modified three-dimensional test at Guru Tegh Bahadur Hospital, Delhi, India. J. Antimicrob. Chemother..

[3] Upadhyay S., Sen M.R., Bhattacharjee A. (2010). Presence of different β-lactamase classes among clinical isolates of *Pseudomonas aeruginosa* expressing AmpC β-lactamase enzyme. J. Infect. Dev. Ctries..

[4] Paterson D.L. (2000). Recommendation for treatment of severe infections caused by *Enterobacteriaceae* producing extended-spectrum β-lactamases (ESBLs). Clin. Microbiol. Infect..

[5] Winokur P.L., Canton R., Casellas J.M., Legakis N. (2001). Variations in the prevalence of strains expressing an extended-spectrum β-lactamase phenotype and characterization of isolates from Europe, the Americas, and the Western Pacific region. Clin. Infect. Dis..

[6] Paterson D.L., Bonomo R.A. (2005). Extended-spectrum β-lactamases: A clinical update. Clin. Microbiol. Rev..

[7] Jacoby G.A. (2009). AmpC β-lactamases. Clin. Microbiol. Rev..

[8] Costa D., Vinue L., Poeta P., Coelho A.C., Matos M., Saenz Y., Somalo S., Zarazaga M., Rodrigues J., Torres C. (2009). Prevalence of extended-spectrum β-lactamase-producing *Escherichia coli* isolates in faecal samples of broilers. Vet. Microbiol..

[9] Kao C.C., Liu M.F., Lin C.F., Huang Y.C., Liu P.Y., Chang C.W., Shi Z.Y. (2010). Antimicrobial susceptibility and multiplex PCR screening of AmpC genes from isolates of *Enterobacter cloacae*, *Citrobacter freundii*, and *Serratia marcescens*. J. Microbiol. Immunol. Infect..

[10] Bush K. (2010). Alarming β-lactamase-mediated resistance in multidrug-resistant *Enterobacteriaceae*. Curr. Opin. Microbiol..

[11] Dai W., Sun S., Yang P., Huang S., Zhang X., Zhang L. (2013). Characterization of carbapenemases, extended spectrum β-lactamases and molecular epidemiology of carbapenem-non-susceptible *Enterobacter cloacae* in a Chinese hospital in Chongqing. Infect. Genet. Evol..

[12] Heller I., Grif K., Orth D. (2011). Emergence of VIM-1-carbapenemase-producing *Enterobacter cloacae* in Tyrol, Austria. J. Med. Microbiol..

[13] Hamada Y., Watanabe K., Tatsuya T., Mezaki K., Takeuchi S., Shimizu T., Kirikae T., Ohmagari N. (2013). Three cases of IMP-type metallo-β-lactamase-producing *Enterobacter cloacae* bloodstream infection in Japan. J. Infect. Chemother..

[14] Mezzatesta M.L., Gona F., Stefani S. (2012). *Enterobacter cloacae* complex: Clinical impact and emerging antibiotic resistance. Future Microbiol..

[15] Oteo J., Hernandez-Almaraz J.L., Gil-Anton J., Vindel A., Fernandez S., Bautista V., Campos J. (2010). Outbreak of vim-1-carbapenemase-producing *Enterobacter cloacae* in a pediatric intensive care unit. Pediatr. Infect. Dis. J..

[16] Paterson D.L. (2006). Resistance in gram-negative bacteria: *Enterobacteriaceae*. Am. J. Infect. Control..

[17] Woodford N., Dallow J.W., Hill R.L., Palepou M.F., Pike R., Ward M.E., Warner M., Livermore D.M. (2007). Ertapenem resistance among *Klebsiella* and *Enterobacter* submitted in the UK to a reference laboratory. Int. J. Antimicrob. Agents.

[18] Huang S., Dai W., Sun S., Zhang X., Zhang L. (2012). Prevalence of plasmid-mediated quinolone resistance and aminoglycoside resistance determinants among carbapeneme non-susceptible *Enterobacter cloacae*. PLoS ONE.

[19] Yan J.J., Hsueh P.R., Ko W.C., Luh K.T., Tsai S.H., Wu H.M., Wu J.J. (2001). Metallo-β-lactamases in clinical *Pseudomonas isolates* in Taiwan and identification of VIM-3, a novel variant of the VIM-2 enzyme. Antimicrob. Agents Chemother..

[20] Yan J.J., Ko W.C., Chuang C.L., Wu J.J. (2002). Metallo-β-lactamase-producing *Enterobacteriaceae* isolates in a university hospital in Taiwan: Prevalence of IMP-8 in *Enterobacter cloacae* and first identification of VIM-2 in *Citrobacter freundii*. J. Antimicrob. Chemother..

[21] Yan J.J., Hsueh P.R., Lu J.J., Chang F.Y., Ko W.C., Wu J.J. (2006). Characterization of acquired β-lactamases and their genetic support in multidrug-resistant *Pseudomonas aeruginosa* isolates in Taiwan: The prevalence of unusual integrons. J. Antimicrob. Chemother..

[22] Tseng S.P., Tsai J.C., Teng L.J., Hsueh P.R. (2009). Dissemination of transposon Tn6001 in carbapenem-non-susceptible and extensively drug-resistant *Pseudomonas aeruginosa* in Taiwan. J. Antimicrob. Chemother..

[23] Lee C.H., Chia J.H., Chu C., Wu T.L., Liu J.W., Su L.H. (2006). In vivo selection of OmpK35-deficient mutant after cefuroxime therapy for primary liver abscess caused by *Klebsiella pneumoniae*. J. Antimicrob. Chemother..

[24] Lee C.H., Chu C., Liu J.W., Chen Y.S., Chiu C.J., Su L.H. (2007). Collateral damage of flomoxef therapy: In vivo development of porin deficiency and acquisition of *bla*_DHA-1_ leading to ertapenem resistance in a clinical isolate of *Klebsiella pneumoniae* producing CTX-M-3 and SHV-5 β-lactamases. J. Antimicrob. Chemother..

[25] Liu Y.F., Yan J.J., Ko W.C., Tsai S.H., Wu J.J. (2008). Characterization of carbapenem-non-susceptible *Escherichia coli* isolates from a university hospital in Taiwan. J. Antimicrob. Chemother..

[26] Yang F.C., Yan J.J., Hung K.H., Wu J.J. (2011). Characterization of ertapenem-resistant *Enterobacter cloacae* in a Taiwanese university hospital. J. Clin. Microbiol..

[27] Yan J.J., Ko W.C., Tsai S.H., Wu H.M., Jin Y.T., Wu J.J. (2000). Dissemination of CTX-M-3 and CMY-2 β-lactamases among clinical isolates of *Escherichia coli* in southern Taiwan. J. Clin. Microbiol..

[28] Yan J.J., Hsueh P.R., Lu J.J., Chang F.Y., Shyr J.M., Wan J.H., Liu Y.C., Chuang Y.C., Yang Y.C., Tsao S.M. (2006). Extended-spectrum β-lactamases and plasmid-mediated AmpC enzymes among clinical isolates of *Escherichia coli* and *Klebsiella pneumoniae* from seven medical centers in Taiwan. Antimicrob. Agents Chemother..

[29] Su P.A., Wu L.T., Cheng K.C., Ko W.C., Chuang Y.C., Yu W.L. (2010). Screening extended-spectrum β-lactamase production in *Enterobacter cloacae* and *Serratia marcescens* using antibiogram-based methods. J. Microbiol. Immunol. Infect..

[30] CLSI (2006). Performance Standards for Antimicrobial Susceptibility Testing.

[31] CLSI (2014). Performance Standards for Antimicrobial Susceptibility Testing.

[32] Kado C.I., Liu S.T. (1981). Rapid procedure for detection and isolation of large and small plasmids. J. Bacteriol..

[33] Rasmussen B.A., Bush K. (1997). Carbapenem-hydrolyzing β-lactamases. Antimicrob. Agents Chemother..

[34] Bellais S., Girlich D., Karim A., Nordmann P. (2002). EBR-1, a novel Ambler subclass B1 β-lactamase from *Empedobacter brevis*. Antimicrob. Agents Chemother..

[35] Poirel L., Nordmann P. (2002). Acquired carbapenem-hydrolyzing β-lactamases and their genetic support. Curr. Pharm. Biotechnol..

[36] Queenan A.M., Bush K. (2007). Carbapenemases: The versatileβ-lactamases. Clin. Microbiol. Rev..

[37] Perez-Perez F.J., Hanson N.D. (2002). Detection of plasmid-mediated AmpC β-lactamase genes in clinical isolates by using multiplex PCR. J. Clin. Microbiol..

[38] Poirel L., Heritier C., Tolun V., Nordmann P. (2004). Emergence of oxacillinase-mediated resistance to imipenem in *Klebsiella pneumoniae*. Antimicrob. Agents Chemother..

[39] Eckert C., Gautier V., Saladin-Allard M., Hidri N., Verdet C., Ould-Hocine Z., Barnaud G., Delisle F., Rossier A., Lambert T. (2004). Dissemination of CTX-M-type β-lactamases among clinical isolates of *Enterobacteriaceae* in Paris, France. Antimicrob. Agents Chemother..

[40] Nüesch-Inderbinen M.T., Hächler H., Kayser F.H. (1996). Detection of genes coding for extended-spectrum SHV β-lactamases in clinical isolates by a molecular genetic method, and comparison with the E test. Eur. J. Clin. Microbiol. Infect. Dis..

[41] Song W., Kim J.S., Kim H.S., Jeong S.H., Yong D., Lee K.M. (2006). Emergence of *Escherichia coli* isolates producing conjugative plasmid-mediated DHA-1 β-lactamase in a Korean university hospital. J. Hosp. Infect..

[42] Bellais S., Aubert D., Naas T., Nordmann P. (2000). Molecular and biochemical heterogeneity of class B carbapenem-hydrolyzing β-lactamases in *Chryseobacterium meningosepticum*. Antimicrob. Agents Chemother..

[43] Stumpf A.N., Roggenkamp A., Hoffmann H. (2005). Specificity of enterobacterial repetitive intergenic consensus and repetitive extragenic palindromic polymerase chain reaction for the detection of clonality within the *Enterobacter cloacae* complex. Diagn. Microbiol. Infect. Dis..

[44] Dice L.R. (1945). Measures of the amount of ecological association between species. Ecology.

[45] Sneath P.H., Sokal R.R. (1973). Numerical Taxonomy. The Principles and Practice of Numerical Classification.

[46] Juan C., Macia M.D., Gutierrez O., Vidal C., Perez J.L., Oliver A. (2005). Molecular mechanisms of β-lactam resistance mediated by AmpC hyperproduction in *Pseudomonas aeruginosa* clinical strains. Antimicrob. Agents Chemother..

[47] Mammeri H., Poirel L., Nordmann P. (2003). In vivo selection of a chromosomally encoded β-lactamase variant conferring ceftazidime resistance in *Klebsiella oxytoca*. Antimicrob. Agents Chemother..

[48] Mohd Khari F.I., Karunakaran R., Rosli R., Tee Tay S. (2016). Genotypic and phenotypic detection of AmpC β-lactamases in *Enterobacter* spp. isolated from a teaching hospital in Malaysia. PLoS ONE.

[49] Papanicolaou G.A., Medeiros A.A., Jacoby G.A. (1990). Novel plasmid-mediated β-lactamase (MIR-1) conferring resistance to oxyimino- and α-methoxy β-lactams in clinical isolates of *Klebsiella pneumoniae*. Antimicrob. Agents Chemother..

[50] Bradford P.A., Urban C., Mariano N., Projan S.J., Rahal J.J., Bush K. (1997). Imipenem resistance in *Klebsiella pneumoniae* is associated with the combination of ACT-1, a plasmid-mediated AmpC β-lactamase, and the loss of an outer membrane protein. Antimicrob. Agents Chemother..

[51] Wang S., Xiao S.Z., Gu F.F., Tang J., Guo X.K., Ni Y.X., Qu J.M., Han L.Z. (2017). Antimicrobial susceptibility and molecular epidemiology of clinical *Enterobacter cloacae* bloodstream isolates in Shanghai, China. PLoS ONE.

[52] Ingti B., Laskar M.A., Choudhury S., Maurya A.P., Paul D., Talukdar A.D., Choudhury M.D., Dhar D., Chakravarty A., Bhattacharjee A. (2017). Molecular and in silico analysis of a new plasmid-mediated AmpC β-lactamase (CMH-2) in clinical isolates of *Klebsiella pneumoniae*. Infect. Genet. Evol..

[53] Yu W.L., Winokur P.L., von Stein D., Pfaller M.A., Wang J.H., Jones R.N. (2002). First description of *Klebsiella pneumoniae* harboring CTX-M β-lactamases (CTX-M-14 and CTX-M-3) in Taiwan. Antimicrob. Agents Chemother..

[54] Wu L.T., Wu H.J., Chung J.G., Chuang Y.C., Cheng K.C., Yu W.L. (2006). Dissemination of *Proteus mirabilis* isolates harboring CTX-M-14 and CTX-M-3 β-lactamases at 2 hospitals in Taiwan. Diagn. Microbiol. Infect. Dis..

[55] Wu L.T., Tsou M.F., Wu H.J., Chen H.E., Chuang Y.C., Yu W.L. (2004). Survey of CTX-M-3 extended-spectrum β-lactamase (ESBL) among cefotaxime-resistant *Serratia marcescens* at a medical center in middle Taiwan. Diagn. Microbiol. Infect. Dis..

[56] Yu W.L., Cheng K.C., Chi C.J., Chen H.E., Chuang Y.C., Wu L.T. (2006). Characterisation and molecular epidemiology of extended-spectrum β-lactamase-producing *Enterobacter cloacae* isolated from a district teaching hospital in Taiwan. Clin. Microbiol. Infect..

[57] Chen C.M., Yu W.L., Huang M., Liu J.J., Chen I.C., Chen H.F., Wu L.T. (2015). Characterization of IS26-composite transposons and multidrug resistance in conjugative plasmids from *Enterobacter cloacae*. Microbiol. Immunol..

