# Supplementary material for: Detection of Plasmid-Mediated β-Lactamase Genes and Emergence of a Novel AmpC (CMH-1) in Enterobacter cloacae at a Medical Center in Southern Taiwan
Source: J Clin Med. 2018 Dec 20;8(1):8. doi: 10.3390/jcm8010008 (PMC6352282; doi:10.3390/jcm8010008)
Supplement: Supplementary file 1 [file jcm-08-00008-s001.pdf]

```
LOCUS       blaCMH-1                1146 bp    DNA     linear   BCT 15-FEB-2012
DEFINITION  Enterobacter cloacae strain 6 class C beta-lactamase CMH-1
            (blaCMH-1) gene, complete cds.
ACCESSION   blaCMH-1
SOURCE      Enterobacter cloacae
ORGANISM    Enterobacter cloacae
            Bacteria; Proteobacteria; Gammaproteobacteria; Enterobacteriales;
            Enterobacteriaceae; Enterobacter; Enterobacter cloacae complex.
REFERENCE   1  (bases 1 to 1146)
  AUTHORS   Yu,W.-L., Lee,M.-F. and Chuang,Y.-C.
  TITLE     A novel plasmid-borne ampC gene (blaCMH-1) in an Enterobacter
            cloacae isolate from southern Taiwan
  JOURNAL   Submitted (15-FEB-2012) Departments of Intensive Care Medicine,
            Chi-Mei Medical Center, No. 901 Zhonghua Rd., YongKang Dist.,
            Tainan, Taiwan 710, Republic of China
FEATURES             Location/Qualifiers
     source          1..1146
                     /organism="Enterobacter cloacae"
                     /mol_type="genomic DNA"
                     /strain="6"
                     /isolation_source="blood"
                     /db_xref="taxon:550"
                     /note="[cultured bacterial source]"
     gene            1..1146
                     /gene="blaCMH-1"
     CDS             1..1146
                     /gene="blaCMH-1"
                     /function="beta-lactamase"
                     /note="class C beta-lactamase CMH-1"
                     /codon_start=1
                     /transl_table=11
                     /product="beta-lactamase CMH-1"
                     /translation="MMTKSLSCALLLSVACSAFAAPMSEKQLADVVERTVTP
                     LMKAQAIPGMAVAVIYQGQPHYFTFGKADVAANKPVTPQTLFELGS
                     VSKTFTGVLGGDAIARKEISLADPVTKYWPELTGKQWQGIRLLDLAT
                     YTAGGLPLQVPDDVTDNASLLRFYQSWQPKWAPGTTRLYANTSIGL
                     FGSLAVKPSGMCFEQAMAERVFKPLKLNHTWINVPHAEEPHYAWGY
                     REGKAVHVSPGMLDAEAYGVKSNVKDMASWVMANMAPETLPQST
                     LQQGIALAQSRYWRVGAMYQGLGWEMLNWPVDAKTVVDGSDNK
                     VALAPLPVAEVNPPAPPVKASWVHKTGSTGGFGSYVAFIPEKQIGIVM
                     LANKSYPNPVRVETAYRILDALQ*"
BASE COUNT        252 a      316 c      334 g      244 t
ORIGIN
        1 atgatgacaa aatccctaag ctgcgcgctg ctgctcagcg ttgcctgctc tgcttttgcc
       61 gcgccgatgt cagaaaaaca gctggctgac gtcgtggaac gtaccgttac gcctctgatg
      121 aaggcgcagg ccatacccgg aatggccgtg gccgtcattt atcagggcca gccacactat
      181 tttactttcg gtaaagcaga cgtcgcggcg aataagcccg tcacgccgca aaccttattt
      241 gagctgggct ccgtcagcaa aaccttcacc ggcgtgctgg gtggcgatgc cattgcccgc
      301 aaagagattt cgctggccga cccggtcacg aaatattggc ctgaattgac gggcaagcag
      361 tggcaaggca ttcgcctgct agacctggca acctataccg caggcggatt gccgttgcag
      421 gtaccggatg atgtcaccga taacgcctct ctgctgcgtt tctaccagtc ctggcagcca
      481 aagtgggccc cgggtaccac gcgtctgtac gccaacacca gcatcggttt gtttggctca
      541 ctggccgtta aaccgtccgg catgtgcttc gagcaggcca tggcggagcg ggtctttaag
      601 cccctgaaac tcaaccatac gtggataaac gttccacacg ctgaagaacc gcactacgca
      661 tggggttatc gtgagggaaa agcggtccac gtttcgcctg gtatgctgga tgcagaagcc
      721 tatggcgtga aatctaacgt caaagatatg gcgagttggg tgatggccaa tatggcacct
      781 gagacactcc cgcagtccac tctgcagcag ggtattgcgc tggcgcagtc tcgctactgg
      841 cgcgtgggtg ccatgtatca agggttaggc tgggagatgc tcaactggcc ggtcgatgcc
      901 aaaaccgtgg tggatggcag cgataataag gtcgcactgg cgccgttgcc ggtcgcagaa
      961 gtgaatcctc cggctccgcc agtaaaagcc tcctgggtgc ataaaacggg ctctacgggt
     1021 gggtttggca gctacgtggc gtttattcct gaaaagcaga tcggtattgt gatgctcgca
     1081 aataaaagct atccgaaccc ggtacgggtg gaaacggctt accgtatcct cgacgcgcta
     1141 cagtaa
//
```

JQ673557

Supplementary Figure1. Complete DNA sequence of *bla*CMH-1 gene (accession number JQ673557).

```
>ref|YP_003611068.1| G class C beta-lactamase [Enterobacter cloacae subsp. cloacae ATCC
13047]
 gb|ADF60119.1| G class C beta-lactamase [Enterobacter cloacae subsp. cloacae ATCC 13047]

Length=381

 GENE ID: 9123062 ampC | class C beta-lactamase
[Enterobacter cloacae subsp. cloacae ATCC 13047] (10 or fewer PubMed links)

 Score =  777 bits (2007),  Expect = 0.0, Method: Compositional matrix adjust.
 Identities = 379/381 (99%), Positives = 379/381 (99%), Gaps = 0/381 (0%)

Query  1    MMTKSLSCALLLSVACSAFAAPMSEKQLADVVERTVTPLMKAQAIPGMAVAVIYQGQPHY  60  Ent. C 6 (CMH-1)
            MM KSLSCALLLSVACSAFAAPMSEKQLADVVERTVTPLMKAQAIPGMAVAVIYQGQPHY
Sbjct  1    MMKKSLSCALLLSVACSAFAAPMSEKQLADVVERTVTPLMKAQAIPGMAVAVIYQGQPHY  60

Query  61   FTFGKADVAANKPVTPQTLFELGSVSKTFTGVLGGDAIARKEISLADPVTKYWPELTGKQ  120
            FTFGKADVAANKPVTPQTLFELGSVSKTFTGVLGGDAIARKEISLADPVTKYWPELTGKQ
Sbjct  61   FTFGKADVAANKPVTPQTLFELGSVSKTFTGVLGGDAIARKEISLADPVTKYWPELTGKQ  120

Query  121  WQGIRLLDLATYTAGGLPLQVPDDVTDNASLLRFYQSWQPKWAPGTTRLYANTSIGLFGS  180
            WQGIRLLDLATYTAGGLPLQVPDDVTDNASLLRFYQSWQPKWAPGTTRLYANTSIGLFGS
Sbjct  121  WQGIRLLDLATYTAGGLPLQVPDDVTDNASLLRFYQSWQPKWAPGTTRLYANTSIGLFGS  180

Query  181  LAVKPSGMCFEQAMAERVFKPLKLNHTWINVPHAEEPHYAWGYREGKAVHVSPGMLDAEA  240
            LAVKPSGM FEQAMAERVFKPLKLNHTWINVPHAEEPHYAWGYREGKAVHVSPGMLDAEA
Sbjct  181  LAVKPSGMRFEQAMAERVFKPLKLNHTWINVPHAEEPHYAWGYREGKAVHVSPGMLDAEA  240

Query  241  YGVKSNVKDMASWVMANMAPETLPQSTLQQGIALAQSRYWRVGAMYQGLGWEMLNWPVDA  300
            YGVKSNVKDMASWVMANMAPETLPQSTLQQGIALAQSRYWRVGAMYQGLGWEMLNWPVDA
Sbjct  241  YGVKSNVKDMASWVMANMAPETLPQSTLQQGIALAQSRYWRVGAMYQGLGWEMLNWPVDA  300

Query  301  KTVVDGSDNKVALAPLPVAEVNPPAPPVKASWVHKTGSTGGFGSYVAFIPEKQIGIVMLA  360
            KTVVDGSDNKVALAPLPVAEVNPPAPPVKASWVHKTGSTGGFGSYVAFIPEKQIGIVMLA
Sbjct  301  KTVVDGSDNKVALAPLPVAEVNPPAPPVKASWVHKTGSTGGFGSYVAFIPEKQIGIVMLA  360

Query  361  NKSYPNPVRVETAYRILDALQ  381
            NKSYPNPVRVETAYRILDALQ
Sbjct  361  NKSYPNPVRVETAYRILDALQ  381
```

Supplementary Figure 2. The *bla*CMH-1 gene has high identities of 99% to a chromosomally intrinsic ampC gene in *Enterobacter cloac*ae ATCC 13047 strain (accession number YP_003611068).

```
>ref|YP_004712370.1| G class C beta-lactamase ACT-9 [Pantoea agglomerans]
 gb|AEI70575.1| G class C beta-lactamase ACT-9 [Pantoea agglomerans]
Length=380

 GENE ID: 10929439 ampC | class C beta-lactamase ACT-9 [Pantoea agglomerans]

 Score =  699 bits (1804),  Expect = 0.0, Method: Compositional matrix adjust.
 Identities = 333/380 (88%), Positives = 354/380 (93%), Gaps = 0/380 (0%)

Query  1    MMTKSLSCALLLSVACSAFAAPMSEKQLADVVERTVTPLMKAQAIPGMAVAVIYQGQPHY  60 EntC-6 (CMH-1)
            M TKSL CALLLS +CS  AAPMSEKQL+DVVERTVTPLMKAQAIPGMAVAVIYQGQPHY
Sbjct  1    MKTKSLCCALLLSTSCSVLAAPMSEKQLSDVVERTVTPLMKAQAIPGMAVAVIYQGQPHY  60

Query  61   FTFGKADVAANKPVTPQTLFELGSVSKTFTGVLGGDAIARKEISLADPVTKYWPELTGKQ  120
            FTFGKADV ANKPVTPQTLFELGS+SKTFTGVLGGDAIAR EISL DPVTKYWPELTGKQ
Sbjct  61   FTFGKADVTANKPVTPQTLFELGSISKTFTGVLGGDAIARGEISLGDPVTKYWPELTGKQ  120

Query  121  WQGIRLLDLATYTAGGLPLQVPDDVTDNASLLRFYQSWQPKWAPGTTRLYANTSIGLFGS  180
            WQGIR+LDLATYTAGGLPLQVPD+VTDN SLLRFYQ WQP+W PGTTRLYAN SIGLFG+
Sbjct  121  WQGIRMLDLATYTAGGLPLQVPDEVTDNTSLLRFYQHWQPQWKPGTTRLYANASIGLFGA  180

Query  181  LAVKPSGMCFEQAMAERVFKPLKLNHTWINVPHAEEPHYAWGYREGKAVHVSPGMLDAEA  240
            LAVKPSGM FEQAM +RVFKPLKL+HTWINVP  EE HYAWGYR+GKA+HVSPGMLDAEA
Sbjct  181  LAVKPSGMNFEQAMTKRVFKPLKLDHTWINVPKEEEAHYAWGYRDGKAIHVSPGMLDAEA  240

Query  241  YGVKSNVKDMASWVMANMAPETLPQSTLQQGIALAQSRYWRVGAMYQGLGWEMLNWPVDA  300
            YGVK+N++DMASW+ ANM P+ L  STL+QGIALAQSRYWRVGAMYQGLGWEMLNWPV+A
Sbjct  241  YGVKTNIQDMASWLKANMNPDALSDSTLKQGIALAQSRYWRVGAMYQGLGWEMLNWPVEA  300

Query  301  KTVVDGSDNKVALAPLPVAEVNPPAPPVKASWVHKTGSTGGFGSYVAFIPEKQIGIVMLA  360
            KTVV+GSDNKVALAPL VAEVNPPAPPVKASWVHKTGSTGGFGSYVAFIPEK++GIVMLA
Sbjct  301  KTVVEGSDNKVALAPLLVAEVNPPAPPVKASWVHKTGSTGGFGSYVAFIPEKELGIVMLA  360

Query  361  NKSYPNPVRVETAYRILDAL  380
            NKSYPNP RVE AYRIL AL
Sbjct  361  NKSYPNPARVEAAYRILSAL  380
```

Supplementary Figure 3. The *bla*CMH-1 gene has 88% identities to a chromosomal ACT-9 gene in *Pantoea agglomerans* (accession number YP_004712370).

```
>emb|CAJ28994.1|  Class C beta-lactamase ACT-2 [Enterobacter asburiae]
Length=381

 Score =  699 bits (1803),  Expect = 0.0, Method: Compositional matrix adjust.
 Identities = 331/380 (87%), Positives = 355/380 (93%), Gaps = 0/380 (0%)

Query  1    MMTKSLSCALLLSVACSAFAAPMSEKQLADVVERTVTPLMKAQAIPGMAVAVIYQGQPHY  60   EntC-6 (CMH-1)
            MMTKSL CALLLS +CS  A PMSEKQLA+VVERTVTPLMKAQAIPGMAVAVIY+GQPHY
Sbjct  2    MMTKSLCCALLLSTSCSVLATPMSEKQLAEVVERTVTPLMKAQAIPGMAVAVIYEGQPHY  61

Query  61   FTFGKADVAANKPVTPQTLFELGSVSKTFTGVLGGDAIARKEISLADPVTKYWPELTGKQ  120
            FTFGKADVAANKPVTPQTLFELGS+SKTFTGVLGGDAIAR EISL DPVTKYWPELTGKQ
Sbjct  62   FTFGKADVAANKPVTPQTLFELGSISKTFTGVLGGDAIARGEISLGDPVTKYWPELTGKQ  121

Query  121  WQGIRLLDLATYTAGGLPLQVPDDVTDNASLLRFYQSWQPKWAPGTTRLYANTSIGLFGS  180
            WQGIR+LDLATYTAGGLPLQVPD+VTDNASLLRFYQ+WQP+W PGTTRLYAN SIGLFG+
Sbjct  122  WQGIRMLDLATYTAGGLPLQVPDEVTDNASLLRFYQNWQPQWKPGTTRLYANASIGLFGA  181

Query  181  LAVKPSGMCFEQAMAERVFKPLKLNHTWINVPHAEEPHYAWGYREGKAVHVSPGMLDAEA  240
            LAVKPSGM +EQA+  RVFKPLKL+HTWINVP AEE HYAWGYR+GKAVHVSPGMLDAEA
Sbjct  182  LAVKPSGMSYEQAITTRVFKPLKLDHTWINVPKAEEAHYAWGYRDGKAVHVSPGMLDAEA  241

Query  241  YGVKSNVKDMASWVMANMAPETLPQSTLQQGIALAQSRYWRVGAMYQGLGWEMLNWPVDA  300
            YGVK+NV+DMASWVM NM P++L  ++L++G+ LAQSRYWRVGAMYQGLGWEMLNWPVDA
Sbjct  242  YGVKTNVQDMASWVMVNMKPDSLQDNSLRKGLTLAQSRYWRVGAMYQGLGWEMLNWPVDA  301

Query  301  KTVVDGSDNKVALAPLPVAEVNPPAPPVKASWVHKTGSTGGFGSYVAFIPEKQIGIVMLA  360
            KTVV+GSDNKVALAPLP  EVNPPAPPV ASWVHKTGSTGGFGSYVAFIPEKQ+GIVMLA
Sbjct  302  KTVVEGSDNKVALAPLPAREVNPPAPPVNASWVHKTGSTGGFGSYVAFIPEKQLGIVMLA  361

Query  361  NKSYPNPVRVETAYRILDAL  380
            NKSYPNP RVE AYRIL AL
Sbjct  362  NKSYPNPARVEAAYRILSAL  381
```

Supplementary Figure 4. The *bla*CMH-1 gene has 87% identities to a chromosomal ACT-2 gene in *Enterobacter asburiae* (accession number CAJ28994).
